# Job satisfaction and retention of health-care providers in Afghanistan and Malawi

**DOI:** 10.1186/1478-4491-12-11

**Published:** 2014-02-17

**Authors:** Linda Fogarty, Young Mi Kim, Hee-Soon Juon, Hannah Tappis, Jin Won Noh, Partamin Zainullah, Aleisha Rozario

**Affiliations:** 1 Jhpiego/USA, an affiliate of Johns Hopkins University, 1615 Thames St., Baltimore, MD, USA; 2 Department of Health, Behavior and Society, Bloomberg School of Public Health, The Johns Hopkins University, 624 N. Broadway, Baltimore, MD, USA; 3 Department of International Health, Bloomberg School of Public Health, The Johns Hopkins University, 615 N. Wolfe St, Baltimore, MD, USA; 4 Department of Healthcare Management, Eulji University, 212 Yangji-dong, Sujeong-gu, Seongnam, Korea; 5 Jhpiego/Afghanistan, an affiliate of Johns Hopkins University, Share-Naw Street 3, House 265, Kabul, Afghanistan

**Keywords:** Afghanistan, Job satisfaction, Malawi, Retention

## Abstract

**Background:**

This study describes job satisfaction and intention to stay on the job among primary health-care providers in countries with distinctly different human resources crises, Afghanistan and Malawi.

**Methods:**

Using a cross-sectional design, we enrolled 87 health-care providers in 32 primary health-care facilities in Afghanistan and 360 providers in 10 regional hospitals in Malawi. The study questionnaire was used to assess job satisfaction, intention to stay on the job and five features of the workplace environment: resources, performance recognition, financial compensation, training opportunities and safety. Descriptive analyses, exploratory factor analyses for scale development, bivariate correlation analyses and bivariate and multiple linear regression analyses were conducted.

**Results:**

The multivariate model for Afghanistan, with demographic, background and work environment variables, explained 23.9% of variance in job satisfaction (F(9,73) = 5.08; *P* < 0.01). However, none of the work environment variables were significantly related to job satisfaction. The multivariate model for intention to stay for Afghanistan explained 23.6% of variance (F(8,74) = 4.10; *P* < 0.01). Those with high scores for recognition were more likely to have higher intention to stay (β = 0.328, *P* < 0.05). However, being paid an appropriate salary was negatively related to intent to stay (β = -0.326, *P* < 0.01). For Malawi, the overall model explained only 9.8% of variance in job satisfaction (F(8,332) = 4.19; *P* < 0.01) and 9.1% of variance in intention to stay (F(10,330) = 3.57; *P* < 0.01).

**Conclusions:**

The construction of concepts of health-care worker satisfaction and intention to stay on the job are highly dependent on the local context. Although health-care workers in both Afghanistan and Malawi reported satisfaction with their jobs, the predictors of satisfaction, and the extent to which those predictors explained variations in job satisfaction and intention to stay on the job, differed substantially. These findings demonstrate the need for more detailed comparative human resources for health-care research, particularly regarding the relative importance of different determinants of job satisfaction and intention to stay in different contexts and the effectiveness of interventions designed to improve health-care worker performance and retention.

## Background

Efforts to improve national and international health indicators, including the Millennium Development Goals, are limited by the quantity and quality of human resources for health (HRH) available to implement lifesaving health-care services [1]. Health-care worker shortages are common globally, but are particularly critical in areas where health indicators are the poorest. Causes of extreme shortages may vary greatly from country to country. They include protracted civil war and unrest, outmigration of trained workers to countries with higher wages and quality of life and a legacy of insufficient government investment in the health-care sector [2,3]. However, there is widespread recognition that, regardless of the cause, extreme shortages of qualified health-care workers present significant barriers to health-care service delivery and that increasing the distribution and retention of health-care workers is critical to improving health system performance [4,5].

The varied causes, but similar consequences of health-care worker shortages are strikingly clear in countries as distinct as Afghanistan and Malawi, where Jhpiego, an affiliate of Johns Hopkins University, has worked with national partners to improve health-care availability and quality for over 10 years. In Afghanistan, many skilled professionals fled the country as refugees, and women’s education was halted under Taliban rule, creating a debilitating shortage of female health-care service providers, who are particularly critical for providing maternal health-care services [6]. In contrast, the reason for health-care worker shortages in Malawi is not forced migration, but economically driven outmigration of health-care workers to high-income countries, with the added service burden of high HIV prevalence rates [7]. Despite such starkly different contexts, the consequences of extreme health-care worker shortages and demands are similar. Both countries report health-care provider-to-patient ratios and maternal and child health indicators that are among the worst in the world [8].

As in many countries where the HRH crisis is most acute, efforts are under way in both Afghanistan and Malawi to address the crisis by improving the planning, development and support of the health-care workforce, including improving workplace conditions to increase health-care worker retention. Since the fall of the Taliban in 2001, the Ministry of Public Health (MoPH) in Afghanistan has steadily worked to rebuild the health-care system in collaboration with donors, multilateral organizations and nongovernmental organizations. From the earliest days of reconstruction, the government has recognized the key role of human resources in a functioning health-care system, developing a standardized service delivery package for primary health care and establishing training programs to address the severe shortage of maternal health-care service providers, particularly midwives and allied health-care workers [9-11]. Between 2004 and 2010, more than 2,350 women graduated from midwifery education programs, thus increasing the number of qualified midwives in the country fivefold from 467 registered with the MoPH in 2002 [12,13]. Shortages of qualified maternal health-care workers remain, however, particularly in more rural and insecure areas [14].

Malawi has had a national HRH plan to address the shortage of health-care workers since the late 1990s. One recent initiative supported by the Ministry of Finance, the UK Department for International Development (DFID) and the Global Fund (implemented from April 2004 to June 2010) offered financial incentives to health-care workers in their posts, recruited health-care workers to fill vacancies, expanded preservice training capacity and strengthened human resources management [15,16]. As a result, from 2004 to 2009, the number of health-care workers increased by 53% [15]. Even with this increase, retention of health-care staff continues to be a problem [16]. The health-care worker shortage in Malawi is still at crisis levels, and high HIV prevalence rates continue to burden the health-care system. The country continues to have one of the lowest provider-to-patient ratios in the world, with only 3 trained health-care providers (doctors, nurses and midwives) per 10,000 population [17].

In both Malawi and Afghanistan, like many other countries struggling with extreme health-care worker shortages, the dearth of trained professionals is further compounded when health-care workers are drawn to more lucrative jobs in other sectors or are not willing to be deployed to remote or insecure areas. A growing number of studies are exploring the factors driving the motivation and retention of health-care workers in developing countries [2,3,18]. In some contexts, job security, recognition and better living conditions have been highlighted as key motivators for placement and retention in remote areas. In other contexts, bundles of interventions that include attention to salary, working conditions and development opportunities have been successful. Studies in Malawi have indicated that managers and midlevel health-care providers are motivated by different factors, but that concerns about salary and career progression are common across all levels of staff [7,19]. At the time of this writing, no research on health-care worker motivation or retention in Afghanistan has been conducted.

This study was designed to describe job satisfaction and intention to stay on the job among primary health-care providers in countries with distinctly different human resources crises and to examine the extent to which the factors affecting health-care worker retention in these two countries are captured by existing literature on the global HRH crisis. Examining the two cases side-by-side provides an opportunity to reconsider the fundamental characteristics underlying job satisfaction. Its results are intended to inform policymakers interested in improving health-care workforce retention in Malawi and Afghanistan, determine the need for further research and serve as an example for stakeholders in other countries that are interested in understanding the extent to which strategies to strengthen the health-care workforce must be tailored to address human resources crises in their own countries.

## Methods

### Study setting

Afghanistan and Malawi were selected as sites for this study because of the clear HRH burdens in both settings and because Jhpiego’s long-standing engagement in health-care sector development in both countries provided an opportunity to incorporate assessments of health-care provider motivation and retention in evaluations of large-scale health-care quality assurance and improvement interventions in each country. Given the impact of the female health-care worker shortage on maternity care in Afghanistan, where cultural norms prohibit male clinicians from providing services to female patients, the study was embedded in an evaluation of a US Agency for International Development (USAID)–funded maternal health-care quality assurance and improvement program at 32 primary health-care facilities in five relatively secure and accessible provinces of Afghanistan (Baghlan, Herat, Jawzjan, Kabul and Takhar). In Malawi, the provider motivation and retention assessment was undertaken within the context of infection prevention–strengthening activities across multiple wards, including (but not exclusively) maternal health care, in 10 regional hospitals. These two groups of providers are critical actors within the HRH crisis of each country.

### Sample and data collection

In these low-resources facilities, providers who were available and not seeing patients were recruited. In Afghanistan, facilities included eight district hospitals, 12 comprehensive health centres and 12 basic health centres. Interviewers recruited a minimum of two reproductive or maternal health-care service providers per facility. In Malawi, interviewers recruited a minimum of two providers per department at each hospital. Providers from seven departments were selected (antenatal care, labour and delivery, family planning, postnatal care, casualty/medical/surgical, operating theatre and waste management). Interviews were conducted in local languages by trained data collectors familiar with the health-care setting. To minimize the potential for social desirability bias, the interviewer explained the purpose, confidentiality and anonymity of the study to each provider before seeking consent to begin the interview. Interviews were conducted face-to-face with clinical health-care providers (medical doctors, nurse midwives, clinical officers and medical assistants) using a standardized, structured questionnaire.

### Ethical considerations

The study received human subjects review and approval from the National Health Sciences Research Committee in Malawi, the Afghan Public Health Institute in Afghanistan and the Western Institutional Review Board in the United States. Informed consent was obtained from all participating health-care providers.

### Measures

The study questionnaires (Additional files 1 & 2) were adapted from the Workplace Climate and Job Satisfaction Survey developed under the USAID-funded Capacity Project and used to assess Kenya’s Emergency Hiring Plan [20]. The instrument elicited respondents’ background information (control variables), features of the work environment (independent variables) and job satisfaction and intent to remain on the job (dependent variables). Background information included age, gender, marital status, having young children, travel time to work and number of years at the current health-care facility.

Features of the work environment were assessed using 17 items covering five areas, namely: resources, performance recognition, financial compensation, training opportunities and safety. Seven questions were asked about their overall opinions of their jobs: job satisfaction (i.e., overall job satisfaction, whether respondent would take the current job if deciding again, whether respondent would recommend a similar job to a friend, whether respondent would choose his or her current health-care provider cadre if he or she could choose any type of job) and intention to stay in the current job as a proxy for actual worker retention (i.e., recently considered switching to another job, recently considered stopping work, plans to stop work in coming year). For each of the items related to work environment, job satisfaction and intent to stay, respondents were asked the following: “Please indicate how much you agree or disagree with the following statements”. Responses were provided on a five-point Likert-type scale, from strongly disagree (1) to strongly agree (5). The questionnaire was pretested before data collection began, and interviewers attended a two-day training program on how to administer the questionnaire.

The following control variables were included: age, gender, having young children, time to travel to work and number of years at the same health-care facility. Based on exploratory data analysis, age was included as a continuous variable for Afghanistan and a dichotomous variable for Malawi (rating 0 being <40 and rating 1 being 40+). Number of years at the same facility was categorized into two groups (0 rating being <2 years and rating 1 being 3+ years). Having young children for whom the health-care providers had to provide care was a dichotomous variable (rating 0 being yes and rating 1 being no). Time required to travel from home to work was categorized into three groups (rating 0 being <30 minutes, rating 1 being 30 to 59 minutes and rating 2 being 1 hour to 3 hours).

### Scale development

Exploratory factor analyses were conducted on the measures of work environment and job satisfaction and worker retention using principal component extraction with a varimax rotation to examine the structure of the responses and determine whether unique patterns of items could be identified. Internal consistency reliability was assessed by using Cronbach’s α coefficient. Table 1 presents the psychometrics of aspects of the work environment and job satisfaction, as well as descriptive statistics (mean ± SD) and the internal reliability of each item. Most of the constructed variables comprised two- or three-item scales; therefore, coefficient α values of 0.50 or 0.60 were considered acceptable [20]. For independent variables, the *sufficient resources* factor encompassed four items (α = 0.63 in Afghanistan; α = 0.59 in Malawi): enough health-care providers, enough support staff, enough drugs and supplies and adequate equipment. Two questions were excluded with low item-to-total correlations (expected to do and overall morale level). The *recognition* factor encompassed three items (α = 0.69 in Afghanistan; α = 0.55 in Malawi): whether health-care providers received constructive feedback from a supervisor, whether they received feedback from a co-worker and whether their contributions were recognized. The *training opportunities* factor encompassed two items (α = 0.57 for both Afghanistan and Malawi). However, the training opportunities factor comprised a different set of variables for each country. For Afghanistan, it comprised two variables: leave time and training provided. For Malawi, it comprised training provided and opportunities to receive training. *Being paid an appropriate salary* was a single item measured as another aspect of work environment. The *safety* factor consisted of three items (α = 0.86 in Afghanistan; α = 0.42 in Malawi): feel safe from physical harm while working, feel safe from physical harm while traveling to work and policies in place to protect workers from harassment. Higher scores indicate more positive experiences with the work environment.

**Table 1:** **Psychometric and bivariate comparisons of work environment and job satisfaction measures**^**a**^

**Construct**	**No. of questions**	**Afghanistan (*N*** **= 87)**		**Malawi (*N*** **= 360)**		***P*-value (*t*-test)**
		**α**	**Mean (±SD)**	**α**	**Mean (±SD)**	
Sufficient resources	4	0.63	3.59 (0.79)	0.59	3.01 (0.92)	<0.01
Recognition	3	0.69	3.46 (1.17)	0.55	3.62 (1.11)	0.25
Paid an appropriate salary	1	N/A	2.99 (1.38)	N/A	1.89 (1.34)	<0.01
Training opportunities	2	0.57	4.05 (1.09)	0.57	3.09 (1.30)	N/A
Safety	3	0.86	3.92 (1.18)	0.42	3.68 (1.00)	0.08
Job satisfaction	4	0.55	3.97 (0.86)	0.72	4.19 (1.02)	<0.05
Intent to stay	3	0.82	2.67 (1.41)	0.54	4.00 (1.13)	<0.01

N/A, not applicable.

^a^With two different questions used to construct training opportunities in Afghanistan and Malawi and calculate the mean differences between two countries.

For dependent variables, principal component factor analysis was conducted on this measure to determine the underlying factor structure, and the factors were rotated using the varimax method. The factor solution was composed of seven items grouped into two factors that accounted for 56.59% of the variance for Afghanistan and 54.36% for Malawi. In Afghanistan, factor 1, intent to stay (eigenvalue = 2.28), accounted for 32.55% of the variance with three items, and factor 2, job satisfaction (eigenvalue = 1.68), accounted for 24.03% of the variance with four items. In Malawi, factor 1, job satisfaction (eigenvalue = 2.69), accounted for 38.47% of the variance, and factor 2, intent to stay (eigenvalue = 1.11), accounted for 15.88% of the variance. The four items were summed together to make a scale of job satisfaction (*n* = 4, α = 0.55 in Afghanistan; 0.72 in Malawi), representing higher scores with higher job satisfaction. The three items were summed together to make a scale of intent to stay (*n* = 3, α = 0.82 in Afghanistan; α = 0.55 in Malawi), with higher scores representing higher intention to stay on the job.

### Statistical analyses

Four sets of analyses were conducted. First, descriptive analyses were performed to provide background information on the sample. Second, bivariate comparisons between the two countries were conducted using *t*-tests. The third set of analyses examined associations between aspects of the work environment and the two dependent variables: job satisfaction and intent to stay in the current job (referred to as *intent to stay*). Pearson correlation analyses were performed to examine the bivariate relationships between the main independent variables (i.e., work environment), the control variables and the two outcomes (job satisfaction and intent to stay). The fourth set of bivariate and multivariate linear regression analyses examined the relationship between multiple aspects of the work environment and each of the dependent variables, job satisfaction and intent to stay. Any variable whose bivariate test had a *P*-value <0.25 was selected for inclusion in the multivariate model [21]. Lowess plots were used to check the linearity of continuous independent and control variables. All analyses were performed with Stata version 11.2 software [22].

## Results

### Sample characteristics

The sample consisted of 87 health-care providers in Afghanistan and 360 health-care providers in Malawi. Sample characteristics for both countries are presented in Table 2. In Afghanistan, the mean age was 33.4 years (SD 7.62), ranging from 20 to 50 years old. All providers except one were female. More than two-thirds (69%) were midwives, 16% were nurses, and 13% were physicians. Four-fifths were married, and more than half had young children. Approximately half travelled to work in less than 30 minutes. About 43% had worked at the same facility less than two years, and the median was three years.

**Table 2:** Sample characteristics of health-care providers in Afghanistan and Malawi

**Characteristics**	**Afghanistan (*N*** **= 87)**	**Malawi (*N*** **= 360)**	***P*-value by χ**^**2**^**/*t*-test**
Age (mean ± SD), range (years)	33.4 ± 7.62, 20 to 50	36.7 ± 11.4, 17 to 75	0.86
Gender			
Male	1 (0.01)	121 (33.7)	<0.001
Female	86 (99.9)	238 (66.3)	
Cadre			
Medical doctor	11 (12.6)	40 (11.1)	N/A
Midwife	60 (69.0)		
Nurse	14 (16.1)		
Midwife/nurse		213 (59.2)	
Support staff/medical assistant	2 (2.3)	47 (13.1)	
Others		60 (16.7)	
Marital status			
Married	69 (80.2)	223 (62.6)	<0.01
Not married	17 (19.8)	133 (37.4)	
Having young children			
Yes	46 (52.9)	263 (73.3)	<0.003
No	41 (47.1)	96 (26.7)	
Time to travel to work			
Less than 30 minutes	40 (46.0)	272 (75.6)	<0.001
30 to 59 minutes	22 (25.3)	68 (18.9)	
1 to 3 hours	25 (28.7)	20 (5.5)	
Type of facility			
District hospital	46 (52.9)	228 (63.3)	N/A
Comprehensive health centre	32 (36.8)		
Basic health centre	9 (10.3)		
Central hospital		25 (6.9)	
Cham-affiliated hospital		107 (29.7)	
Years in the facility			
Less than or equal to 2 years	37 (42.5)	138 (39.3)	0.63
3 or more years	50 (57.5)	213 (60.7)	

N/A, not applicable.

In Malawi, the mean age was 36.7 years (SD ±11.40), ranging from 17 to 75 years old. Nearly two-thirds of respondents were female. More than one-half (59.2%) were nurses or midwives, and 11% were medical doctors. About three-fourths were married and had young children. About three-quarters travelled to work in less than 30 minutes. Approximately two-fifths (39.3%) reported having worked at the same facility less than two years, and the median was three years. There were significant differences in gender, marital status, having young children and travel time between Afghanistan and Malawi.

### Job satisfaction and intent to stay

As shown in Table 1, there was no statistically significant difference in job satisfaction between study participants in Afghanistan and Malawi. However, intent to stay on the job was significantly higher in Malawi (4.00 on a scale of 1 to 5) than in Afghanistan (2.67). There were no significant differences in job satisfaction or intent to stay by cadre or by type of facility (data not shown).

### Bivariate relationships between control, independent and dependent variables

Table 3 presents zero-order correlations between variables in Afghanistan (lower-half matrix) and Malawi (upper-half matrix). In Afghanistan, job satisfaction was positively correlated with two aspects of work environment measured at a *P*-value <0.05 (i.e., Pearson’s *r* = 0.24 for being paid an appropriate salary; Pearson’s *r* = 0.22 for training opportunities), whereas intent to stay was negatively correlated with being paid an appropriate salary (Pearson’s *r* = -0.22). Recognition was marginally correlated to intent to stay (Pearson’s *r* = 0.19, *P* = 0.08). Years on the job was negatively correlated to intent to stay (*r* = -0.23, *P* < 0.05). Having young children was positively correlated to job satisfaction (Pearson’s *r* = 0.23, *P* < 0.05), and travel time to work was negatively correlated to job satisfaction (*r* = -0.42, *P* < 0.01). All five measures of work environment were significantly correlated to each other, with the exception that recognition was not correlated with sufficient resources or safety. Job satisfaction was not correlated with intent to stay.

**Table 3:** **Zero-order correlations between background, work environment, job satisfaction and intent to stay among Malawi health-care providers (*N*** **= 360) (in upper half) and Afghanistan health-care providers (*N*** **= 87) (in lower half)**^**a**^

**Factors**	**Age**	**Years at facility**	**Marital status**	**Having children**	**Time to travel**	**Sufficient resources**	**Recognition**	**Training**	**Appropriate pay**	**Safety**	**Job satisfaction**	**Intent to stay**
Age	**–**	0.47**	-0.24**	-0.33**	0.12*	-0.07	0.20**	0.07	-0.03	0.02	0.12*	0.17**
Years at facility	0.26*	**–**	-0.23**	-0.26**	0.09^+^	-0.01	0.13*	0.04	-0.08	0.11*	-0.04	0.06
Marital status	0.37**	0.16	**–**	0.52**	-0.15**	0.06	-0.04	-0.01	-0.01	0.12*	0.07	-0.03
Having children	0.14	0.17	0.46**	**–**	-0.13*	0.04	-0.15**	0.07	0.04	0.06	0.01	-0.17**
Time to travel	0.10	0.02	0.06	-0.08	**–**	-0.12*	0.06	-0.20**	-0.01	-0.18**	-0.08	0.02
Sufficient resources	-0.23*	-0.01	0.10	0.09	-0.23*	**–**	0.07	0.25**	0.17**	0.29**	0.09^+^	0.11*
Recognition	-0.21^+^	0.11	-0.17	0.08	-0.10	0.02	**–**	0.26**	0.10^+^	0.23**	0.19**	0.14**
Training	-0.22*^+^	0.02	0.16	0.03	-0.23*	0.51**	0.10	**–**	0.12*	0.36**	0.25**	0.15**
Appropriate pay	-0.14	-0.08	-0.17	0.18	-0.25*	0.19^+^	0.38**	0.17	**–**	0.13*	0.11*	0.12**
Safety	-0.09	-0.06	-0.01	0.21*	-0.18^+^	0.40**	0.02	0.21^+^	0.27*	**–**	0.20**	0.02
Job satisfaction	-0.08	0.05	0.06	0.23*	-0.42**	0.14	0.07	0.22*	0.24*	0.12	**–**	0.38**
Intent to stay	-0.17	-0.23*	-0.03	-0.13	0.09	-0.14	0.19^+^	-0.05	-0.22*	-0.11	-0.07	**–**

^+^*P* < 0.10, *P < 0.05, **P < 0.01. Married (yes = 1, no = 0), having young children (yes = 1, no = 0) and years at the same facility (3+ years = 1, ≤2 years = 0).

^a^Upper half is for Malawi, and lower half is for Afghanistan.

In Malawi, job satisfaction was positively correlated with all five measures of work environment at *P* < 0.05: recognition (Pearson’s *r* = 0.19), training opportunities (Pearson’s *r* = 0.25), being paid an appropriate salary (Pearson’s *r* = 0.11) and safety scores (Pearson’s *r* = 0.20). The sufficient resources rating was marginally related to job satisfaction (Pearson’s *r* = 0.09, *P* = 0.09). Intent to stay was also significantly correlated with higher sufficient resources (Pearson’s *r* = 0.11), recognition (Pearson’s *r* = 0.14), training opportunities (Pearson’s *r* = 0.15) and appropriate pay (Pearson’s *r* = 0.12), but was not related to safety. Age was correlated with job satisfaction (Pearson’s *r* = 0.12) and intent to stay (Pearson’s *r* = 0.17, *P* < 0.01). Having young children was negatively correlated with intent to stay (Pearson’s *r* = -0.17, *P* < 0.01). All five measures of work environment were significantly correlated to each other, with the exception that recognition was not correlated with sufficient resources. Job satisfaction and intent to stay were significantly correlated with each other (Pearson’s *r* = 0.38, *P* <0.01).

In both countries, age and years at the same facility were related (Pearson’s *r* = 0.26, *P* < 0.05 in Afghanistan; Pearson’s *r* = 0.47, *P* < 0.01 in Malawi). Because marital status and having young children were highly correlated (Pearson’s *r* = 0.46, *P* < 0.01 in Afghanistan; Pearson’s *r* = 0.52, *P* < 0.01 in Malawi), only the construct having children was included in multivariate analysis.

### Multivariate linear regression of job satisfaction and worker retention

Table 4 presents the results of linear regression of job satisfaction and intent to stay in Afghanistan. In bivariate analysis, time to travel to work, having young children, cadre, paid an appropriate salary and training opportunities were significantly associated with job satisfaction (*P* < 0.05). In multivariate analysis, those who had longer travel time to work (i.e., 1 to 3 hours) had lower job satisfaction than those whose travel time to work was less than 30 minutes (β = -0.665). Cadre was associated with job satisfaction: Nurses or assistant nurses had lower job satisfaction than medical doctors (β = -0.511). However, none of the work environment variables were related to job satisfaction. The overall model explained 23.9% of variance in job satisfaction (F(9,73) = 5.08, *P* < 0.01).

**Table 4:** **Multiple linear regression model with unstandardized β coefficients (±SE) for job satisfaction and intent to stay among Afghanistan health-care providers (*N*** **= 83)**

**Characteristics**	**Job satisfaction**		**Intent to stay**	
	**Bivariate**	**Multivariate**	**Bivariate**	**Multivariate**
Age	-0.009 (0.014)	-0.005 (0.013)	-0.031 (0.020)^++^	-0.026 (0.020)
Years at the same facility				
(0 to 2 years = reference value)	0.079 (0.191)	N/A	-0.643 (0.295)*	-0.613 (0.288)*
Having young children	0.395 (0.183)*	0.222 (0.173)	-0.354 (0.298)^++^	0.034 (0.280)
Time to travel to work				
(<30 minutes = reference value)				
30 to 59 minutes	-0.728 (0.229)**	-0.557 (0.289)^+^	0.518 (0.345)	N/A
1 to 3 hours	-0.808 (0.184)**	-0.665 (0.207)**	0.240 (0.371)	
Cadre				
(Medical doctor = reference value)				
Community midwife	-0.360 (0.182)^+^	-0.312 (0.207)	0.435 (0.463)	0.774 (0.379)^+^
Nurse/assistant nurse	-0.537 (0.259)*	-0.511 (0.254)*	0.674 (0.550)^++^	1.098 (0.500)*
Sufficient resources	0.157 (0.113)^++^	-0.058 (0.115)	-0.250 (0.170)^++^	-0.292 (0.190)
Recognition	0.055 (0.073)	N/A	0.226 (0.127)^+^	0.328 (0.126)*
Paid an appropriate salary	0.149 (0.063)*	0.049 (0.068)	-0.223 (0.109)*	-0.326 (0.118)**
Training opportunities	0.171 (0.075)*	0.085 (0.095)	-0.061 (0.125)	N/A
Safety	0.086 (0.088)	N/A	-0.136 (0.123)	N/A
*R*^2^		0.239		0.236

N/A: not applicable.

^++^0.25 < *P* < 0.10, ^+^*P* < 0.10, *P < 0.05, **P < 0.01**.**

In bivariate analysis, years on the job and paid an appropriate salary were related to intention to stay (*P* < 0.05). Recognition was marginally associated with intent to leave (*P* = 0.06). In multivariate analysis, those who stayed at the same facility more than three years had lower intent to stay than those who stayed at the same facility less than two years (β = -0.613, *P* < 0.05). Cadre was related to intent to stay: Nurse/nurse assistants were more likely to have intent to stay than medical doctors (β = 1.098, *P* < 0.05). Two aspects of work environment, recognition and paid an appropriate salary, were associated with intent to stay: Those with high scores for recognition were more likely to have higher intent to stay (β = 0.328, *P* < 0.05). Interestingly, being paid an appropriate salary was negatively related to intent to stay (β = -0.326, *P* < 0.01. The overall model explained 23.6% of variance in intention to stay (F(8,74) = 4.10, *P* < 0.01).

Table 5 presents the results of linear regression of job satisfaction and intent to stay in Malawi. In bivariate analysis, four aspects of the work environment were significantly associated with job satisfaction: recognition, paid an appropriate salary, training opportunities and safety. The categorical age variable was also related to job satisfaction. In multivariate analysis, training opportunities was significantly related to job satisfaction: Those who had more training opportunities had higher job satisfaction (β = 0.123, *P* < 0.05). Safety was marginally associated with job satisfaction (*P* = 0.067). Those older than 40 years of age reported higher job satisfaction than those younger than 40 years old. The overall model explained 9.8% of variance in job satisfaction (F(8,332) = 4.19, *P* < 0.01).

**Table 5:** **Multiple linear regression model with unstandardized β coefficients (±SE) for job satisfaction and intent to stay among Malawi providers (*N*** **= 360)**

	**Job satisfaction**		**Intent to stay**	
**Characteristics**	**Bivariate**	**Multivariate**	**Bivariate**	**Multivariate**
Age (≤0 years = reference value)	0.242 (0.114)*	0.262 (0.126)*	0.377 (0.122)**	0.275 (0.139)*
40+				
Years at the same job				
(0 to 2 years = reference value)	-0.073 (0.108)^++^	-0.231 (0.121)^+^	0.143 (0.123)^++^	-0.037 (0.139)
3+				
Gender (0 = male)				
Female	0.114 (0.117)	N/A	0.013 (0.123)	N/A
Having young children	0.018 (0.123)	N/A	-0.440 (0.142)**	-0.373 (0.167)*
Time to travel to work (0 = <30 min)				
30-59 min	-0.027 (0.132)	0.077 (0.130)	0.051 (0.158)	N/A
1 to 3 hours	-0.430 (0.248)^+^	-0.101 (0.243)	0.079 (0.261)	
Cadre (0 = Medical doctor)				
Midwife/nurse	-0.151 (0.200)	N/A	0.041 (0.185)	-0.040 (0.188)
Supporting staff/medical assistant	-0.188 (0.167)		0.115 (0.189)	0.020 (0.200)
Others	0.064 (0.155)		0.288 (0.148)^+^	0.132 (0.157)
Sufficient resources	0.097 (0.056)^+^	-0.001 (0.059)	0.140 (0.016)*	0.087 (0.069)
Recognition	0.172 (0.05)**	0.086 (0.053)	0.144 (0.056)*	0.054 (0.065)
Paid an appropriate salary	0.083 (0.034)*	0.042 (0.038)	0.097 (0.040)*	0.081 (0.045)^+^
Training opportunities	0.195 (0.041)**	0.123 (0.046)**	0.127 (0.047)**	0.101 (0.051)*
Safety	0.207 (0.054)**	0.112 (0.060)^+^	0.024 (0.062)	N/A
*R*^2^		0.098		0.091

N/A: not applicable.

^++^0.25 < *P* < 0.10, ^+^*P* < 0.10, *P < 0.05, **P < 0.01.

In bivariate analysis, four aspects of the work environment (i.e., sufficient resources, recognition, paid an appropriate salary and training opportunities) were associated with intent to stay. Age and having young children were significantly related to intent to stay. In multivariate analysis, one aspect of work environment—training opportunities—was associated with intent to stay: Those with high scores for training opportunities were more likely to have higher intent to stay (β = 0.101, *P* < 0.05). Paid an appropriate salary was marginally associated with intent to stay (*P* = 0.072). In addition, those older than 40 years of age reported higher intent to stay than those younger than 40 years old (β = 0.275, *P* < 0.05). Those without young children reported lower intent to stay than those with young children (β = -0.373, *P* < 0.05). The overall model explained 9.1% of variance in intent to stay (F(10,330) = 3.57, *P* < 0.01).

## Discussion

Simultaneously examining two demographically similar samples of health-care providers in two very different settings—Malawi and Afghanistan—provides a new lens for understanding what makes health-care providers satisfied and what makes them want to leave their jobs. Under this lens, characteristics considered fundamental to job satisfaction, such as receiving an appropriate salary, can be seen in a new light.

First, although health-care providers in both Afghanistan and Malawi reported that they were quite satisfied with their jobs, the predictors of satisfaction and the extent to which those predictors explained variation in job satisfaction differed substantially between the two study settings. The linear regression results for Afghanistan, which included demographic, background and work environment variables, explained nearly one-fourth of the variance in job satisfaction. For Malawi, the same categories of variables—demographic and background variables and all work environment variables—explained only one-tenth of the variation in job satisfaction. Variables found to significantly contribute to the model for Malawi were different from those in the Afghanistan model. In Malawi, those who reported that they were recognized for their work and those who had more training opportunities were more satisfied with their jobs, whereas in Afghanistan only time spent traveling to work was independently significant. It is possible that facility-associated factors had greater influence on staff satisfaction in Malawi because the sample was clustered in fewer facilities, with greater likelihood of shared experiences and influences than in Afghanistan.

Second, though the average respondent in Malawi intended to stay in his or her position, the average respondent in Afghanistan did not. Predictors of intent to stay and the extent to which those predictors explained variation in intention also differed substantially between the two study settings. Background information, demographics and work environment variables were better predictors of intention to stay for providers in Afghanistan than for those in Malawi, and the specific indicators were different for the two countries. In Afghanistan, those who had been on the job less time, those who were nurses, those who reported being recognized and those who were less likely to report that their salary was appropriate were more likely to intend to stay in their current positions. For providers in Malawi, however, those who were older, did not have young children and received training opportunities were more likely to intend to stay on the job. In Malawi, younger health-care providers may entertain the idea of possibilities that they see others taking advantage of, such as emigrating for better opportunities for their young families, whereas those opportunities are less available to the female health-care providers in our study living in Afghanistan. The unexpected finding that providers in Afghanistan who did not feel that their salary was appropriate were more intent on staying in their jobs calls for further, possibly qualitative, research to better understand the reasons underlying job retention in this case.

In general, findings from Afghanistan and Malawi are consistent with previous studies that examined job satisfaction of health-care workers in low-, middle- and high-income countries. For respondents in both Afghanistan and Malawi, those who received training opportunities and felt safe from harm at work and traveling to work were more likely to report that they were paid an appropriate salary. In addition, study results in Malawi showed that compensation and training opportunities were both strongly correlated to higher job satisfaction and intent to stay. These results are in line with findings of previous studies that demonstrate that managers and midlevel health-care providers are sometimes motivated by different factors, but that concerns about salary and career progression are common across all levels of staff [3,7,19].

The differences in findings from Afghanistan and Malawi are also consistent with those reported in the literature. Multicountry studies of health-care worker job satisfaction have revealed interesting variations between countries, suggesting that macro-level cultural, economic and political factors, such as labour policies and work culture and expectations might significantly shape an individual’s attitudes towards his or her work [24,25]. This study also found variations in the factors related to job satisfaction and intention to stay across countries. For example, in Malawi, workers who had been at their facilities longer were more likely to have received greater recognition, specifically constructive feedback or recognition for doing good work. However, the opposite was true in Afghanistan; those newer at the facility were more likely to have received constructive feedback. This could be a function of the how “constructive feedback” and “recognition” were interpreted by study participants, could simply be an artefact of how the terms were translated in each country or could be due to other culture- and context-specific differences. Similarly, in Afghanistan, only one factor, salary, was related to job satisfaction and intention to stay, whereas in Malawi, four of the five factors measured were related to job satisfaction and intention to stay. This difference could be a function of the smaller sample size in Afghanistan or could be due to other culture- and context-specific differences, such as differences in expectations of the conditions and benefits a job should provide or levels of inequities in salary within the health-care sector [26]. For example, on the one hand, it appears that job satisfaction is a more complex construct in the Malawi setting, is influenced by more factors and, at least in this sample, is incompletely explained by those health-care workforce indicators typically considered to be important. On the other hand, the construct of job satisfaction in Afghanistan seemed to be a simpler concept that was better explained by a small subset of the factors studied here, particularly “time spent traveling to work”. The fact that even salary was not a significant predictor of job satisfaction may be a function of women in Afghanistan being relatively new to the workforce, having been forbidden from studying and working during the years of Taliban rule. In contrast, those in Malawi who reported that they were recognized for their work and those who had more training opportunities were more satisfied with their jobs, which is what is expected based on previous research. As women become more empowered in Afghanistan to pursue greater opportunities in the workplace, their expectations of and demands in the workplace may become more diverse.

These findings demonstrate the need for more detailed comparative HRH research, particularly on context-specific determinants of job satisfaction and intention to stay, as well as on the effectiveness of interventions designed to improve health-care worker performance and retention. Most job satisfaction data are derived from small-scale surveys conducted with a single category of health-care worker in one country. There have been few job satisfaction studies conducted to formally compare countries or different health-care worker cadres, and even fewer have evaluated both [27,28]. Comparative research could help shed light on the essential underpinnings of the construct of job satisfaction while helping to identify the needs of health-care workers in specific contexts and informing the design of more effective HRH interventions. Also, similarly to health-care worker surveys, in our study we did not investigate whether differences in expressed job satisfaction and intention to stay had any real impact on health-care worker performance or retention. Further research is needed to explain some of the associations observed and to investigate their significance for health-care service delivery. In addition, though there has been increased interest among researchers and policymakers in identifying and implementing effective solutions to address HRH shortages in remote and rural areas in recent years, the current evidence base available to guide policymakers on adoption and adaptation of specific retention strategies remains quite limited [18,29-31]. Suggested interventions to improve job satisfaction have been extrapolated mostly from the organizational factors known to be associated with job satisfaction, including caseload, remuneration, physical working conditions, supervision and leadership. More attention also needs to be given to developing interventions and strategies that directly address the factors found to influence provider satisfaction and then to conducting rigorous intervention research to evaluate the effectiveness of these interventions on health-care worker performance and retention in various contexts [25].

Interpretation of our study findings is subject to certain limitations. First, the samples were not designed as nationally representative samples. Second, the samples were not designed to facilitate direct comparison of a parallel group of health-care workers in Afghanistan and Malawi; rather, the samples were designed to capture a group of health-care providers determined to be of particular interest in the context of the HRH crisis of each country. That said, the two groups were similar in terms of some demographic characteristics. Participants were health-care professionals who, on average, were in their mid-30s, and most had been working in the same facility a fair amount of time (on average more than six years). There were minor, but statistically significant differences between participants in the two countries in terms of marital status, having young children and travel time to health-care facilities. One notable exception is that in Afghanistan almost all participants were women (because of the maternal health focus and the cultural norms in Afghanistan that restrict male providers from serving female clients). Third, the studies presented herein were small components of larger studies and did not collect detailed data on all the myriad individual, organizational and contextual determinants. Therefore, the available variables explained only a small proportion of the variation in job satisfaction and intention to leave in the multiple regression models. Finally, like many other studies of health-care worker satisfaction and retention, this analysis is based on cross-sectional rather than longitudinal data and therefore cannot be used to infer causal linkages between satisfaction and intention to stay or actual turnover.

## Conclusions

Health-care worker satisfaction and intention to stay on the job are highly dependent on the local context. We have shown differences in the levels of job satisfaction and intention to stay on the job between different groups of health-care workers in Afghanistan and Malawi, as well as both similarities and differences in the determinants of health-care worker satisfaction in each country, that are consistent with previous studies from other low-, middle- and high-income countries. These findings demonstrate the need for more detailed comparative HRH research, particularly on the relative importance of different determinants of job satisfaction and intention to stay in different contexts and on the effectiveness of interventions designed to improve health-care worker performance and retention.

## Abbreviations

DFID: UK Department for International Development; HRH: Human Resources for Health, Global Health Workforce Alliance/World Health Organization; MoPH: Ministry of Public Health of Afghanistan; USAID: US Agency for International Development.

## Competing interests

The authors declare that they have no competing interests.

## Authors’ contributions

LF led the interpretation of results and writing and revision of the manuscript. YMK designed the study, served as the Principal Investigator and contributed to the writing and revision of the manuscript. HSJ conducted the analysis and contributed to the writing and revision of the manuscript. HT contributed to the interpretation of results and the writing and revision of the manuscript. JWN conducted the literature review and contributed to the first draft of the manuscript. PZ participated in the design and implementation of the study in Afghanistan and contributed to the interpretation of study findings and revision of the manuscript. AR participated in the design and implementation of the study in Malawi and contributed to the interpretation of study findings and writing of the manuscript. All authors read and approved the final manuscript.

## Supplementary Material

Additional file 1 Jhpiego/Afghanistan Provider Interview Guide. Click here for file

Additional file 2 Jhpiego/Malawi Provider Interview Guide. Click here for file
