# Supplementary material for: Job satisfaction and retention of health-care providers in Afghanistan and Malawi
Source: Hum Resour Health. 2014 Feb 17;12:11. doi: 10.1186/1478-4491-12-11 (PMC3930828; doi:10.1186/1478-4491-12-11)
Supplement: Additional file 1 — Jhpiego/Afghanistan Provider Interview Guide. [file 1478-4491-12-11-S1.doc]

**
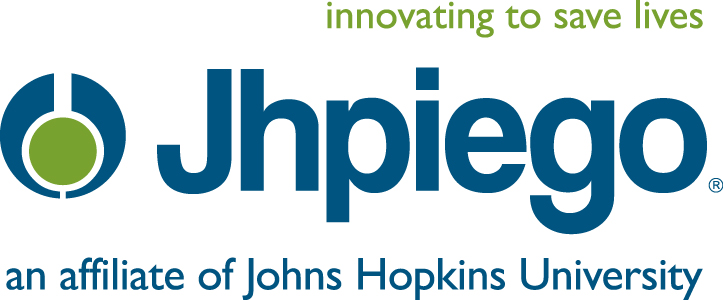
**

| **Provider Study ID Number:** |
| --- |

**PROVIDER INTERVIEW GUIDE***

*Adapted from the Workplace Climate and Job Satisfaction Survey developed under the USAID-funded Capacity Project

**Evaluation of Quality Assurance and Improvement in Reproductive and Maternal Health**

**in Non-Remote, Secure, and USAID-funded Provinces of Afghanistan**

1. Facility ID Number:
2. Provider ID Number:
3. Date of observation:______ / _______ / _______

DD / MM / YYYY

1. Interviewer ID:
2. Start Time: ______:______ (hours: minutes)
3. End Time: ______:______ (hours: minutes)

**SECTION 1: PROVIDER INFORMATION**

101. Respondent’s Cadre ***[Circle one]***

[1] Assistant Nurse

[2] Auxiliary Midwife

[3] Midwife

[4] Nurse

[5] Community Midwife

[6] MD – Obstetrician/Gynecologist

[7] MD Surgeon

[8] MD – Specialist Other __________

[9] Other ___________

102. How long have you worked at this health facility? _________years _________months

103. How long have you ever worked as a [cadre], including your work in this health facility? __________years _________ months

104. In which service do you work in this facility? ***[Circle all that apply.]***

[1] Antenatal Care (ANC) service

[2] Maternity/Labor and Delivery

[3] Family Planning (FP)

[4] Postpartum Care (PNC) service

[5] Casualty/Medical/Surgical service

[6] Other _______________________________________

105. How old were you on your last birthday? _________ years old

106. Sex: [1] Male [2] Female

107. What language do you speak at home?

[1] Dari/Hazara

[2] Pashto

[3] Uzbek

[4] Turkmen

[5] Other ______________________

108. Are you currently married? [1] Yes [2] No

109. Do you have young children for whom you must provide care? **[1]** Yes **[2]** No

110. How long have you lived at your current home? __________ years

111. How much time does it take for you to travel from your home to this health facility? ***[Circle one.]***

[1] Less than 30 minutes

[2] 30–59 minutes

[3] 1 hour to 3 hours

[4] More than 3 hours

112. Do your spouse or dependents live in the same house in which you are living currently? **[1]** Yes **[2]** No

113. How long do you and/or your family intend to stay at the current house? ________ years

**SECTION 2: ASPECTS OF THE WORK ENVIRONMENT**

Now I would like to ask you about specific aspects of the work environment. Please indicate how much you agree or disagree with the following statements. The response categories are “Strongly Disagree,” “Somewhat Disagree,” “Neither Disagree Nor Agree/Unsure,” “Somewhat Agree,” and “Strongly Agree.” *[Please circle the appropriate number corresponding to the response given.]*

| **Q** | **Question** | ***Strongly***  ***Disagree*** | ***Somewhat Disagree*** | ***Neither Disagree Nor Agree/ Unsure*** | ***Somewhat Agree*** | ***Strongly Agree*** |
| --- | --- | --- | --- | --- | --- | --- |
|  | ***Work duties*** |  |  |  |  |  |
| 201 | Currently, you know what you are *expected* to do on the job. | 1 | 2 | 3 | 4 | 5 |
| 202 | Currently, there are enough *health providers* in this facility to offer services adequately to this community. | 1 | 2 | 3 | 4 | 5 |
| 203 | Currently, there are enough *support staff* in this facility to perform the tasks that need to be done. | 1 | 2 | 3 | 4 | 5 |
| 204 | Currently, there are enough *drugs and supplies* for you to do your job well (i.e., gloves, needles, bandages, oxytocin…). | 1 | 2 | 3 | 4 | 5 |
| 205 | Currently, there is *equipment* you need to do your job well and efficiently (i.e., ultrasound, x-ray, blood pressure cuffs). | 1 | 2 | 3 | 4 | 5 |
| 206 | Currently, overall, the *morale* level in your department is good. | 1 | 2 | 3 | 4 | 5 |
|  | ***Supervision and recognition*** |  |  |  |  |  |
| 207 | In the past year, you have received *constructive feedback* from a supervisor; so you know how well you are performing tasks. | 1 | 2 | 3 | 4 | 5 |
| 208 | In the past year, you have received *constructive feedback* from a co-worker; so you know how well you are performing tasks. | 1 | 2 | 3 | 4 | 5 |
| 209 | In the past year, you have been *recognized* for doing good work, either as an individual or as part of a team. | 1 | 2 | 3 | 4 | 5 |
|  | ***Compensation, benefits, and professional development*** |  |  |  |  |  |
| 210 | You are currently paid a *salary* that is appropriate for the work you do. | 1 | 2 | 3 | 4 | 5 |
| 211 | In the past year, you have received your salary at the time that it is due to be paid to you. | 1 | 2 | 3 | 4 | 5 |
| 212 | In the past year, you have been able to take *leave time* that is due to you, when you wish to take it. | 1 | 2 | 3 | 4 | 5 |
| 213 | *Training* is provided, or paid for, by this facility in the skills that are critical for you to succeed. | 1 | 2 | 3 | 4 | 5 |
| 214 | Opportunities to receive training are distributed *fairly*. | 1 | 2 | 3 | 4 | 5 |
|  | ***Safety*** |  |  |  |  |  |
| 215 | You feel safe from *physical harm* when you are *working* in this facility. | 1 | 2 | 3 | 4 | 5 |
| 216 | You feel safe from physical harm when you are *traveling* from your home to this facility. | 1 | 2 | 3 | 4 | 5 |
| 217 | Policies are in place in this facility to protect workers from any type of *harassment.* | 1 | 2 | 3 | 4 | 5 |
|  | ***Fulfillment*** |  |  |  |  |  |
| 218 | Your job is rewarding. | 1 | 2 | 3 | 4 | 5 |
| 219 | Your job is stressful. | 1 | 2 | 3 | 4 | 5 |
| 220 | Your work is important to this facility. | 1 | 2 | 3 | 4 | 5 |
| 221 | You feel isolated in your work. | 1 | 2 | 3 | 4 | 5 |
| 222 | Your work as [cadre] is *valued* by the community. | 1 | 2 | 3 | 4 | 5 |
| 223 | You lack confidence in some of your clinical skills. | 1 | 2 | 3 | 4 | 5 |
| 224 | You feel overworked. | 1 | 2 | 3 | 4 | 5 |
| 225 | Your work has *a positive impact* on the health of people in the community served by this health facility. | 1 | 2 | 3 | 4 | 5 |

**SECTION 3: SUPERVISION**

301. In the past six months, how many times were you supervised?

302. What was done during the supervision? *[Mark with X to all mentioned.]*

|  | **Question** | **Yes (1)** | **No (0)** |
| --- | --- | --- | --- |
| 302a | Check facility records and reports? |  |  |
| 302b | Observe your work? |  |  |
| 302c | Give you feedback on your performance? |  |  |
| 302d | Give you updates on administrative or technical issues related to your work? |  |  |
| 302e | Discuss any problems you had? |  |  |
| 302f | Help resolve problems that you or other staff raised? |  |  |

**SECTION 4: JOB SATISFACTION AND RETENTION**

Now I would like to ask about your overall opinions of your job. Please indicate how much you agree or disagree with the following statements. The response categories are “Strongly Disagree,” “Somewhat Disagree,” “Neither Disagree Nor Agree/Unsure,” “Somewhat Agree,” and “Strongly Agree.” *[Please circle the appropriate number corresponding to the response given.]*

| **Q** | **Item** | ***Strongly***  ***Disagree*** | ***Somewhat Disagree*** | ***Neither Disagree Nor Agree/ Unsure*** | ***Somewhat Agree*** | ***Strongly Agree*** |
| --- | --- | --- | --- | --- | --- | --- |
| 401 | If you had to decide all over again whether to take your current job or not, you would take it. | 1 | 2 | 3 | 4 | 5 |
| 402 | You would recommend working in a job like yours at your facility to a friend. | 1 | 2 | 3 | 4 | 5 |
| 403 | If you had a choice to take any type of job you wanted, you would still choose to be a [cadre]. | 1 | 2 | 3 | 4 | 5 |
| 404 | Overall, you are satisfied with your job. | 1 | 2 | 3 | 4 | 5 |
| 405 | In the past year, you have considered switching to another job as a [cadre] in a different facility. | 1 | 2 | 3 | 4 | 5 |
| 406 | In the past year, you have considered stopping your work as a [cadre], or doing something else other than work as a [cadre]. | 1 | 2 | 3 | 4 | 5 |
| 407 | In the coming year, you plan to stop working in this health facility. | 1 | 2 | 3 | 4 | 5 |

**SECTION 5: QUALITY IMPROVEMENT AND ASSURANCE**

501. Did quality improvement/assurance activities happen at this facility? [1] Yes [2] No

502. Have you and your facility been involved in the process of quality improvement/assurance? [1] Yes [2] No

503. In the past years, did you receive any training related to delivery of reproductive health services or infection prevention?

[1] Yes [2] No

504. In the past year, have you received training in the following area?

| [1] Antenatal Care (ANC)  [2] Postnatal Care (PNC) | [3] Family Planning (FP)  [4] Labor/Delivery |
| --- | --- |

**SECTION 6: PERCEIVED REPRODUCTIVE HEALTH SERVICE QUALITY**

Now I would like to ask your opinion on the quality of reproductive health services and infection prevention practices at this facility. The response categories are “Very Poor,” “Poor,” “Neither Poor Nor Good/Unsure,” “Good,” and “Very Good.”

How would you rate the quality of care at this health facility of…? *[Please circle the appropriate number corresponding to the response given.]*

|  | ***Very Poor*** | ***Poor*** | ***Neither Poor Nor Good/Unsure*** | ***Good*** | ***Very Good*** |
| --- | --- | --- | --- | --- | --- |
| 601. Family Planning Services? | 1 | 2 | 3 | 4 | 5 |
| 602. Antenatal Care? | 1 | 2 | 3 | 4 | 5 |
| 603. Delivery Care/Maternity? | 1 | 2 | 3 | 4 | 5 |
| 604. Postpartum Care? | 1 | 2 | 3 | 4 | 5 |

605. Over the past year, has the **quality** of services in your work area at this facility improved?

[1] Yes [2] No

606. Over the past year, has the availability of drugs and supplies in your work area stayed the same, improved, or worsened?

Stayed the same (1)  Improved (2)  Worsened (3)

**Thank you for taking the time to be interviewed.**
