# Supplementary material for: Job satisfaction and retention of health-care providers in Afghanistan and Malawi
Source: Hum Resour Health. 2014 Feb 17;12:11. doi: 10.1186/1478-4491-12-11 (PMC3930828; doi:10.1186/1478-4491-12-11)
Supplement: Additional file 2 — Jhpiego/Malawi Provider Interview Guide. [file 1478-4491-12-11-S2.doc]

**
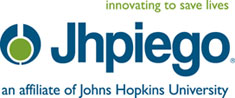
**

**Malawi Provider Job Satisfaction Questionnaire**

**RH and IP PQI Evaluation[[1]](#footnote-2)**

**Interview Information**

q100). Interviewer ID#: _____________

Interview Date: q101). Month __________ q102). Day ____ q103). Year ________

*Tick (√) if the following has occurred:*

__ Provider or interviewer signed the two copies of Consent Form

__ Provider kept one copy, Interviewer kept 2nd copy in a separate folder from this questionnaire

**Facility Information**

q200). Name of Health Facility: __________________________________________

q201). Type of Health Facility *[Circle one.]*

| [1] District Hospital  [2] Central Hospital  [3] CHAM-affiliated hospital |  |
| --- | --- |

q202). Province/Region: __________________________________________

q203). District: __________________________________________

q204). City/Village: __________________________________________

**Provider Information**

q300). Respondent’s Cadre [corresponds to list in Afghan HMIS] *[Circle one.]*

| **Malawi:**  [1] Registered Nurse/Midwife  [2] Enrolled Nurse/Midwife  [3] Nursing/Midwifery Technician  [4] Medical Assistant  [5] Clinical Officer  [6] Medical Officer  [7] MD – Obstetrician/Gynecologist  [8] MD Surgeon  [9] MD – Specialist Other  [10] Medical Doctor [MD] General  [11] Lab Technician/Technologist  [12] Pharmacist/Pharmacy Technologist or Technician  [13] Support Staff  [14] Other (specify) __________________ |  |
| --- | --- |

q301–302). How many months and/or years have you ever worked as a [cadre], including your work in this health facility? (Please add all periods of employment to arrive at total number of years and months. If less than one year, only enter months. If one year or more, enter years and months.)

q301). Number of months: ________________ q302). Number of years: ______________

q303–304). What month and year did you begin working at this health facility?

q303). Month: ________________ q304). Year: ______________

q305). In which part of the facility do you work: *[Circle all that apply.]*

[1] ANC service delivery area

[2] Maternity/Labor and Delivery ward

[3] Family Planning outpatient clinic

[4] Postpartum Care service delivery area

[5] Casualty/Medical/Surgical department

[6] Operating Theatre

[7] Waste Disposal Unit

q306). How much time does it take for you to travel from your home to this health facility? *[Circle one.]*

[1] Less than 30 minutes

[2] 30–59 minutes

[3] 1 hour to 3 hours

[4] More than 3 hours

q307). In what year were you born? ______________

q308). Sex: [1] Male [2] Female

q309). What language do you speak at home? ____________________

q310). Are you currently married?

**[1]** Yes **[2]** No

q311). Do you have young children for whom you must provide care?

**[1]** Yes **[2]** No

q312). In which district do you currently reside? ____________________________________

q313). How long have you lived in that district? (If less than one year, only enter months. If one year or more, enter years and months.)

a) Number of months _________ and/or b) Number of years __________

| **If answer to question # 310, OR # 311 is *yes*, ask the following questions;**  **otherwise, skip to question # 400.** |
| --- |

q314). Do your spouse or dependents reside in the same district where you currently live?

**[1]** Yes **[2]** No

q 315) Do your spouse or dependents reside in the same district where you currently work?

**[1]** Yes **[2]** No

**Aspects of the Work Environment**

Now I would like to ask you about specific aspects of the work environment. Please indicate how much you agree or disagree with the following statements. The response categories are “Strongly Disagree,” “Somewhat Disagree,” “Neither Disagree Nor Agree/Unsure,” “Somewhat Agree,” and “Strongly Agree.”

| Q | Question | *Strongly*  *Disagree* | *Somewhat Disagree* | *Neither Disagree Nor Agree/ Unsure* | *Somewhat Agree* | *Strongly Agree* |
| --- | --- | --- | --- | --- | --- | --- |
|  | ***Work duties*** |  |  |  |  |  |
| q400). | Currently, you know what you are *expected* to do on the job. | 1 | 2 | 3 | 4 | 5 |
| q401). | Currently, there are enough *health providers* in this facility to offer services to this community. | 1 | 2 | 3 | 4 | 5 |
| q402). | Currently, there are enough *support staff* in this facility to perform the tasks that need to be done. | 1 | 2 | 3 | 4 | 5 |
| q403). | Currently, there are enough *drugs and supplies* for you to do your job well (i.e., gloves, needles, bandages, oxytocin…). | 1 | 2 | 3 | 4 | 5 |
| q404). | Currently, there is *equipment* you need to do your job well and efficiently (i.e., ultrasound, x-ray, blood pressure cuffs). | 1 | 2 | 3 | 4 | 5 |
| q405). | Currently, overall, the *morale* level in your department is good. | 1 | 2 | 3 | 4 | 5 |
|  | ***Supervision and recognition*** |  |  |  |  |  |
| q406). | In the past year, you have received *constructive feedback* from a supervisor; so you know how well you are performing tasks. | 1 | 2 | 3 | 4 | 5 |
| q407). | In the past year, you have received *constructive feedback* from a co-worker; so you know how well you are performing tasks. | 1 | 2 | 3 | 4 | 5 |
| q408). | In the past year, you have been *recognized* for doing good work, either as an individual or as part of a team. | 1 | 2 | 3 | 4 | 5 |
| q409). | ***Compensation, benefits, and professional development*** |  |  |  |  |  |
| q410). | You are currently paid a *salary* that is appropriate for the work you do. | 1 | 2 | 3 | 4 | 5 |
| q411). | In the past year, you have received your salary at the time that it is due to be paid to you. | 1 | 2 | 3 | 4 | 5 |
| q412). | In the past year, you have been able to take *leave time* that is due to you, when you wish to take it. | 1 | 2 | 3 | 4 | 5 |
| q413). | Training is provided, or paid for, by this facility in the skills that are critical for you to succeed. | 1 | 2 | 3 | 4 | 5 |
| q414). | Opportunities to receive training are distributed fairly. | 1 | 2 | 3 | 4 | 5 |
|  | ***Safety*** |  |  |  |  |  |
| q415). | You feel safe from *physical harm* when you are *working* in this facility. | 1 | 2 | 3 | 4 | 5 |
| q416). | You feel safe from physical harm when you are *traveling* from your home to this facility. | 1 | 2 | 3 | 4 | 5 |
| q417). | There are policies in place in this facility to protect workers from any type of harassment. | 1 | 2 | 3 | 4 | 5 |
|  | ***Fulfillment*** |  |  |  |  |  |
| q418). | Your job is rewarding. | 1 | 2 | 3 | 4 | 5 |
| q419). | Your job is stressful. | 1 | 2 | 3 | 4 | 5 |
| q420). | Your work is important to this facility | 1 | 2 | 3 | 4 | 5 |
| q421). | You feel isolated in your work. | 1 | 2 | 3 | 4 | 5 |
| q422). | Your work as [cadre] is *valued* by the community. | 1 | 2 | 3 | 4 | 5 |
| q423). | You lack confidence in some of your clinical skills. | 1 | 2 | 3 | 4 | 5 |
| q424). | You feel overworked. | 1 | 2 | 3 | 4 | 5 |
| q425). | Your work has *a positive impact* on the health of people in the community served by this health facility. | 1 | 2 | 3 | 4 | 5 |

**Supervision**

*In the past six months, how many times did your supervisor…?[Please enter the number in the box for each question.*]

|  | Question | Enter Number of Times or 98=Don’t Know |
| --- | --- | --- |
| q500). | Check facility records and reports? |  |
| q501). | Observe your work? |  |
| q502). | Give you feedback on your performance? |  |
| q503). | Give you updates on administrative or technical issues related to your work? |  |
| q504). | Discuss any problems you had? |  |
| q505). | Help resolve problems that you or other staff raised? |  |

**Job Satisfaction/Retention**

Now I would like to ask about your overall opinion of your job. Please indicate how much you agree or disagree with the following statements. The response categories are “Strongly Disagree,” “Somewhat Disagree,” “Neither Disagree Nor Agree/Unsure,” “Somewhat Agree,” and “Strongly Agree.” *[Please circle.]*

| Q | Item | *Strongly*  *Disagree* | *Somewhat Disagree* | *Neither Disagree Nor Agree/ Unsure* | *Somewhat Agree* | *Strongly Agree* |
| --- | --- | --- | --- | --- | --- | --- |
| q600). | If you had to decide all over again whether to take your current job or not, you would take it. | 1 | 2 | 3 | 4 | 5 |
| q601). | You would recommend working in a job like yours at your facility to a friend. | 1 | 2 | 3 | 4 | 5 |
| q602). | If you had a choice to take any type of job you wanted, you would still choose to be a [cadre]. | 1 | 2 | 3 | 4 | 5 |
| q603). | Overall, you are satisfied with your job. | 1 | 2 | 3 | 4 | 5 |
| q604). | In the past year, you have considered switching to another job as a [cadre] in a different facility. | 1 | 2 | 3 | 4 | 5 |
| q605). | In the past year, you have considered stopping your work as a [cadre], or doing something else other than work as a [cadre]. | 1 | 2 | 3 | 4 | 5 |
| q606). | In the coming year, you plan to stop working in this health facility. | 1 | 2 | 3 | 4 | 5 |

**Exposure to PQI Interventions**

q700). Are you aware of any efforts to improve the quality of health services at this facility?

**[1]** Yes **[2]** No

q701). Are you aware of any performance and quality improvement assessment tools in use at this facility?

**[1]** Yes **[2]** No

q702). Are you familiar with any of these quality assessment tools? [Interviewer: Show respondent the tools]

[1] Yes [2] No *Go to question 721*

q703). Have you participated in the process of improving the quality of health services and/or infection prevention practices at this facility using the assessment tools I just showed you?

**[1]** Yes **[2]** No *Go to question 721*

*q704–709) Did you participate in..? [Please circle response for e*ach.]

|  | Yes | No | Unsure | Skip |
| --- | --- | --- | --- | --- |
| q704). Baseline Assessment | 1 | 2 | 9 | 99 |
| q705). Follow-up Assessment(s) | 1 | 2 | 9 | 99 |
| q706). Gap Analysis | 1 | 2 | 9 | 99 |
| q707). Developing Action Plans based on gaps | 1 | 2 | 9 | 99 |
| q708). Improving Health Services/Addressing gaps identified | 1 | 2 | 9 | 99 |
| q709). Other (specify)_____________________ | 1 | 2 | 9 | 99 |

q710–718). For which types of services at this health facility did you participate in using the quality improvement process?

|  | Yes | No | Unsure |
| --- | --- | --- | --- |
| q710). Family Planning services? | 1 | 2 | 9 |
| q711). Antenatal Care? | 1 | 2 | 9 |
| q712). Maternity/Labor and Delivery services? | 1 | 2 | 9 |
| q713). Postpartum Care? | 1 | 2 | 9 |
| q714). Well baby/children under-five services? | 1 | 2 | 9 |
| q715). Tuberculosis Care? | 1 | 2 | 9 |
| q716). Medical/Casualty/Surgery services? | 1 | 2 | 9 |
| q717). Waste Disposal? | 1 | 2 | 9 |
| q718). Other (Specify): | 1 | 2 | 9 |

q719). Were the results of the quality assessments used in your work area?

[1] Yes [2] No *Go to question 721*

q720). If so, how were the results used in your work area?

_____________________________________________________________________________

______________________________________________________________________________

_____________________________________________________________________________

q721). In the past three years, did you receive any training related to delivery of reproductive health services or infection prevention?

[1] Yes [2] No

q722). What was the topic of the training(s)? *[Circle all that apply over the last three years.]*

[1] Focused Antenatal Care/Antenatal Care

[2] Essential Maternal and Newborn Care

[3} Emergency Obstetric and Newborn Care

[4] Infection Prevention

[5] Postpartum Care for mother and/or newborn

[6] Pediatric Care—IMCI

[7] Pediatric Care—well baby/immunization

[8] Tuberculosis

[9] Family Planning

[10] Drug and Supply Management

[11] Other (specify): _______________________________________

**Perceived Reproductive Health Service Quality**

Now I would like to ask your opinion on the quality of reproductive health services and infection prevention practices at this facility. The response categories are “Very Poor,” “Poor,” “Neither Poor Nor Good/Unsure,” “Good,” and “Very Good.” How do you rate the quality of care of…? *[Please circle response for each.]*

|  | *Very Poor* | *Poor* | *Neither Poor Nor Good/Unsure* | *Good* | *Very Good* |
| --- | --- | --- | --- | --- | --- |
| q800). Family Planning services? | 1 | 2 | 3 | 4 | 5 |
| q801). Antenatal Care? | 1 | 2 | 3 | 4 | 5 |
| q802). Delivery Care/Maternity? | 1 | 2 | 3 | 4 | 5 |
| q803). Postpartum Care? | 1 | 2 | 3 | 4 | 5 |
| q804). Infection Prevention practices in general? | 1 | 2 | 3 | 4 | 5 |

q805). Over the past year, has the **quality** of services in your work area at this facility improved?

[1] Yes [2] No

q806). Why is that the case? Please explain your answer:

_____________________________________________________________________________________

_____________________________________________________________________________________

_____________________________________________________________________________________

q807). Over the past year, has the availability of drugs and supplies in your work area stayed the same, improved, or worsened?

Stayed the same (1)  Improved (2)  Worsened (3)

q808). Why is that the case? Please explain your answer:

_____________________________________________________________________________________

_____________________________________________________________________________________

_____________________________________________________________________________________

**Thank you for taking the time to be interviewed.**

1. Adapted from the Workplace Climate and Job Satisfaction Survey developed under the USAID Capacity Project [↑](#footnote-ref-2)
